# “Right to recommend, wrong to require”- an empirical and philosophical study of the views among physicians and the general public on smoking cessation as a condition for surgery

**DOI:** 10.1186/s12910-017-0237-2

**Published:** 2018-01-08

**Authors:** Joar Björk, Niklas Juth, Niels Lynøe

**Affiliations:** 1 Stockholm Centre for Healthcare Ethics (CHE), LIME, Karolinska Institutet, Tomtebodavägen 18 A, 171 77 Stockholm, Sweden; 2 Department of Research and Development, Region Kronoberg, Sigfridsvägen 5, 352 57 Växjö, Sweden

**Keywords:** Smoking, General practitioners, Orthopedic surgeons, Public health ethics, Responsibility for health, Physician patient interaction, Preventive medicine, Smoking cessation

## Abstract

**Background:**

In many countries, there are health care initiatives to make smokers give up smoking in the peri-operative setting. There is empirical evidence that this may improve some, but not all, operative outcomes. However, it may be feared that some support for such policies stems from ethically questionable opinions, such as paternalism or anti-smoker sentiments. This study aimed at investigating the support for a policy of smoking cessation prior to surgery among Swedish physicians and members of the general public, as well as the reasons provided for this.

**Methods:**

A random sample of general practitioners and orthopaedic surgeons (*n* = 795) as well as members of the general public (*n* = 485) received a mail questionnaire. It contained a vignette case with a smoking 57-year old male farmer with hip osteoarthritis. The patient had been recommended hip replacement therapy, but told that in order to qualify for surgery he needed to give up smoking four weeks prior to and after surgery. The respondents were asked whether making such qualifying demands is acceptable, and asked to rate their agreement with pre-set arguments for and against this policy.

**Results:**

Response rates were 58.2% among physicians and 53.8% among the general public. Of these, 83.9% and 86.6%, respectively, agreed that surgery should be made conditional upon smoking cessation. Reference to the peri-operative risks associated with smoking was the most common argument given. However, there was also strong support for the argument that such a policy is mandated in order to achieve long term health gains.

**Conclusions:**

There is strong support for a policy of smoking cessation prior to surgery in Sweden. This support is based on considerations of peri-operative risks as well as the general long term risks of smoking. This study indicates that paternalistic attitudes may inform some of the support for peri-operative smoking cessation policies and that at least some respondents seem to favour a “recommendation strategy” vis-à-vis smoking cessation prior to surgery rather than a “requirement strategy”. The normative reasons speak in favour of the “recommendation strategy”.

**Electronic supplementary material:**

The online version of this article (10.1186/s12910-017-0237-2) contains supplementary material, which is available to authorized users.

## Background

Smoking is a leading cause of ill health worldwide. According to the World Health Organization, one out of ten deaths worldwide is attributable to tobacco smoking [1]. In addition to bringing about untimely death, smoking also contributes to a number of other health related problems, among these peri-operative complications. This has been extensively studied in orthopaedics, where smoking has been associated with a moderate risk increase in such end-points as peri-operative infections, impaired wound healing, arthrodesis, and reduced success of implant surgery [2–4]. For instance, in a recent review of smoking cessation and bone healing current smokers were 37% less likely to be healed and had 2.2 times higher rate of infections two years after open tibia fractures as compared to patients with no smoking history [5]. The same review showed that the mean time for fracture union was 32 weeks for smokers and 26 weeks for non-smokers [5]. There is also evidence from randomized controlled trials that smoking cessation causes a decrease in some, but not all post-operative complications [6, 7]. Furthermore, some but not all studies have found that the timing of smoking cessation prior to surgery affects outcome in a dose response fashion, i.e. longer duration of smoking cessation gives a more beneficial outcome [5, 8, 9].

This has led to initiatives within health care to make smokers cut down or stop smoking prior to elective surgery [10]. Such pre-surgery smoking cessation schemes have varied in the extent and kind of assistance offered (nicotine replacement therapy, varenicline and/or buproprione therapy, behavioural counselling or combined interventions) [8]. Furthermore, the schemes have varied regarding the extent to which smoking cessation is merely recommended, or made a prerequisite for surgery. These strategies may be conceptualized as strategies of “smoking cessation recommendation” versus “smoking cessation requirement”, respectively. Thus in the following, a “recommendation” strategy will refer to a pre-operative scheme where smoking patients are encouraged to stop smoking, but if they refuse to try or try but fail to do so they are still accepted for surgery. On the other hand, a “requirement” strategy will refer to a scheme where failure to comply means the patient is not accepted for surgery. Moreover, “requirement” strategies may be implemented in either of two ways: as “requirement for attempt” strategies (the patient is accepted for surgery only if s/he has made a qualified attempt at giving up smoking, for instance by participating in a smoking cessation programme), or as “requirement for cessation” strategies (the patient is only accepted for surgery if s/he has actually succeeded to stop smoking). Assessing whether the patient has actually succeeded to stop smoking could be done for instance by urine cotinine testing or measurement of carbon monoxide in exhaled breath [11].

There is currently a strong push in society to counteract smoking, and for physicians to address patients’ smoking status as well as intervene whenever possible against smoking [12, 13]. The Swedish National Board of Health and Welfare strongly endorses smoking cessation prior to surgery, but makes no comment on whether they favour a “recommendation” or “requirement” strategy. However, the National Board names “the offering of qualified anti-smoking counselling and nicotine replacement therapy to smokers prior to surgery” a top priority [14]. The National Board cites health problems directly associated with the surgical procedure, such as those mentioned above, as the main reason for this, but in a side passage also refers to “positive long term effects” of smoking cessation [14]. It is not clear whether this refers to risks associated with the specific surgical intervention, or to the general health risks posed by smoking (unrelated to surgery). However, some have argued that the many negative long term effects of tobacco smoking, surgery or no surgery, in themselves constitute a reason to demand or encourage smoking cessation prior to surgery. For instance, in a Cochrane review authors Thomson et al. mention the view that “the preoperative period might be a window for smoking intervention” [7]. This phrasing seems to indicate that an intended goal is smoking cessation per se, rather than just smoking cessation related in time and risk to the specific surgery. The same message is put more succinctly in a meta-analysis of life style modification programmes in intensive care, where the patients are claimed to be in a “teachable moment” [15].

Various rationales may inform individual or institutional desires to intervene against smoking. Most obviously, the focus may be on patients’ (expressed or assumed) best interest. This argument is, however, not unproblematic from an anti-paternalistic point of view. Famously, John Stuart Mill disapproved of paternalism [16], claiming that the only legitimate reason to limit the actions of any individual is to prevent harm to others. However, preventing smoking, it may be argued, is to prevent harm to others. Indeed, smoking may harm third party directly as in environmental tobacco smoke or indirectly as when non-smokers have to pay increased tax or insurance fees to cover smokers’ health care costs. Thus, it is not hard to construct an argument for policies against smoking based on harm reduction. In another tradition, proponents of the *luck egalitarianism* school of thought have argued that patients who are themselves culpable for their disease, for instance smokers developing lung cancer, should – by virtue of this culpability – receive lower prioritization [17–19]. Such “responsibility principle” reasoning in prioritization is controversial [20]. Nonetheless, empirical studies have shown some support for this thought among physicians [21, 22], and thus it may be that negative attitudes towards self-induced disease make up another reason to favour smoking cessation programmes. Finally, negative attitudes to smoking unrelated to disease causality, i.e. focusing on for instance aesthetics, the smell of tobacco or group prejudice, may also motivate those that support programmes of smoking cessation [23].

In addition to the *ethical* issues raised by such attitudes, which will be investigated in the ‘Discussion’ section below, it is worth noting that reasoning according to the “responsibility principle”, as well as harbouring negative attitudes towards smoking/smokers clash with health care *legislation* in for instance Sweden which maintains that health care should be provided to all without regard to previous life conduct [24]. Obviously, that does not mean that no Swedish physicians harbour such attitudes; instead, studies suggest that some do [25]. Some studies have also shown support for paternalistic and stigmatizing attitudes among subsets of physicians [25–27]. As these attitudes are not in line with official norms, they may not be clearly expressed by the physicians. Still they can inform the physician’s behaviour, in ways the physician may or may not be aware of. One way in which this could happen has been labelled “value impregnation of factual aspects” [25]. Thus a physician who is opposed to, for instance, terminal sedation may exaggerate (factual) aspects that disfavour terminal sedation in an actual case, thereby biasing the decision against this treatment option.

In the light of this, the present study was set up to examine two separate issues of ethical relevance. First, we wanted to investigate the *empirical* question of whether Swedish physicians and members of the general public support making elective surgery conditional upon smoking cessation before surgery, and what reasons they give in support for this. Such empirical investigation serves the purpose of surveying the land to assess how popularly accepted a particular practise is. Presumably, the general acceptance of the public as well as the relevant profession is important for the successful implementation of health policies. Second, we wanted to address the *normative* ethical question of whether the reasons stated are ethically defensible. More to the point, we wanted to examine whether Swedish physicians and members of the general public express paternalistic, responsibility-based or stigmatizing attitudes to smokers when they reason about smoking cessation before elective surgery.

## Methods

### The questionnaire

The study was based upon a quantitative and qualitative analysis of written responses to a short vignette. The case presented in the vignette featured a smoking 57-year old farmer with hip osteoarthritis. The patient had been recommended hip arthroplasty, but told that in order to qualify he needed to join a smoking cessation programme before surgery in order to minimize the risk of complications. However, the patient refused to do this, claiming that for him smoking constituted an important source of positive life quality. We consciously chose a vignette “patient” of working age as work status is sometimes considered a strong reason to offer medical interventions. We further designed the vignette so that the disease in question should not be too uncommon, and where questions of increase in life expectancy would be deemed relevant. The respondents were asked to respond to the following statement (henceforth: the main statement): it is right, in a case such as this, to make the elective surgical procedure conditional upon the patient’s stopping smoking 4 weeks prior to and 4 weeks after surgery. The response options were: agree completely, agree to a large extent, disagree to a large extent, and disagree completely. They were also invited to provide comments to explain their judgment (see Table 3 below).

Some follow-up questions were supplied to assess the respondents’ reasons for their judgment. First, respondents were asked to rate their agreement with several pre-set arguments *pro et contra* making surgery conditional upon smoking cessation. Here, the respondents were able to agree to sub-arguments supporting or rejecting their stance in response to the main statement. Thus, what resulted was an assessment of the overall attitude as well as a mapping of agreement with different sub-arguments from both sides of the discussion. The response options were the same as above. In all analyses the first two response options were collapsed to “agree” and the last two were collapsed to “disagree” (see below). Respondents were also asked to state which of these pre-set arguments they considered most important for their overall judgment. The respondents were also given the opportunity to provide their own arguments in addition to the pre-set arguments. Last, the respondents were asked whether their own trust in health care would be affected if it were made standard procedure to make surgery conditional upon smoking cessation in cases such as this (response options: my trust would decrease/my trust would not be influenced/ my trust would increase). Those who claimed that their trust would increase or decrease were classified as *value-influenced* (in this context), and those who claimed their trust would not be affected were classified as *value-neutral* (in this context). The rationale for this dichotomisation was the assumption that those who said their trust would decrease or increase thereby expressed an evaluation of the act in question (i.e. to make surgery conditional upon smoking cessation). Thus we assumed that any physician who stated that making surgery conditional upon smoking cessation found this act good or desirable, and conversely. Those whose own trust would not be influenced would be interpreted as finding the decision ethically neutral. The question about trust in health care should accordingly be understood as a surrogate marker for personal preferences which are not necessarily in accordance with the official values as endorsed by ethical principles and health care law. For the questionnaire, see the “Additional file 1” section.

### Participants

The study group consisted of a random sample of 400 orthopaedic surgeons and 400 General Practitioners (GPs). This sample of physicians was drawn from a commercial database (Cegedim/Stockholm) with participants from all over Sweden. As comparison group we used 499 individuals from the general public randomly selected from the Stockholm tax registry.

### Analysis

The results were presented as proportion with 95% confidence interval (CI). Chi-2 test was used to analyse differences in answering patterns between the studied groups. Logistic regression analysis was performed in order to study associations between the dichotomous main outcome variable and the independent variables that might influence the outcome. A *p*-value <0.05 was considered statistically significant, as well as confidence interval not overlapping each other. Odds Ratio (OR) with 95% confidence intervals were calculated when estimating associations between whether or not it was right to condition surgery and what would happen with physicians’ own trust in health care. The data were registered and analysed using the Epi-info software 6.04 as well as IBM SPSS v. 23. When analysing the comments, we used content analysis [28], focusing on the respondents’ stated reasons in support of their judgment of whether to support or reject making surgery conditional upon smoking cessation prior to surgery. In giving examples of the manifest content in these comments, an effort has been made to present the variety among meaning units, rather than the relative frequency of content matter. Thus, the quotes representing each category are chosen to illustrate varying rather than conforming manifest content - see Table 3.

### Ethics

All respondents were informed about the study’s purpose and voluntary nature in a simple, comprehensible language. The respondents were offered no incentive to participate.

## Results

### Background data

The questionnaire was sent to 800 individuals in the physician group, but 5 questionnaires were returned by the mail service due to unknown address. Out of the remaining 795 physicians 463 responded, resulting in a response-rate of 58.2%. Of these, 232 were orthopaedic surgeons, 196 general practitioners and 35 belonged to other specialities. The recruitment of physicians other than orthopaedic surgeons and GPs was unintentional, resulting from physicians having changed specialities after inclusion in the commercial database used. As these physicians were registered in the database as GPs at the point of entry into the database, they were analysed together with the GPs even though they had subsequently changed medical speciality.

In the comparison group of 499 representatives from the general public, 14 questionnaires were returned by the mail service due to unknown address, leaving 485 possible respondents in the group. Of these, 261 responded resulting in a response-rate of 53.8% please see Table 1.

**Table 1:** Shows background data

	Orthopaedic surgeons:	GPs + others:	General public:
Response rate (total number)	58.4% (232)	58% (231)	53.8% (261)
Median age (range)	54 (33–80)	59 (30–77)	53 (17–81)
Sex (male/female)	84.1%/15.9%	55.4%/44.6%	41%/59%
Proportion current smokers (total number)	0.4% (1)	1.3% (3)	8.3% (21)

### Agreement with the main statement and with pre-set arguments

The majority among physicians as well as members of the general public agreed with the main statement that in cases like this, surgery should be made conditional upon smoking cessation 4 weeks prior to and 4 weeks after surgery. For response patterns - see Table 2. There was no significant difference in answering patterns across categories of sex or age among physicians or the general public. Apart from the well-known uneven sex balance among orthopaedic surgeons, the physician groups were thus considered similar enough to be treated as one group in all subsequent analyses.

**Table 2:** Shows agreement with the main statement and with the pre-set arguments *pro et contra* this statement

		All physicians:		General Public:	
		Proportion agreed (*n*)	Proportion found this argument most important	Proportion agreed (*n*)	Proportion found this argument most important
Response to main statement (“It is right, in a case such as this, to make surgery conditional upon smoking cessation four weeks prior to and after surgery”)		83.9% (386)	*Not applicable*	86.6% (226)	*Not applicable*
Arguments *pro* the main claim	*Because of risk for complications due to smoking*	94.2% (436)	73.4% (246)	93.5% (244)	59.8% (116)
	*Because it is in the patient’s own interest to stop smoking altogether*	65.9% (302)	4.8% (16)	72.0% (185)	10.3% (20)
	*(Respondent’s own argument)*	16.2% (75)	0.9% (3)	14.6% (38)	2.1% (4)
Arguments *contra* the main claim	*Because the patient, if well informed, should be allowed to decide for himself*	39.4% (181)	8.1% (27)	50% (128)	11.9% (23)
	*Because smoking is important to this patient’s life quality*	32.5% (150)	7.2% (24)	37.1% (96)	7.7% (15)
	*(Respondent’s own argument)*	17.8% (82)	4.2% (14)	19.1% (50)	5.7% (11)

Among all physicians as well as among members of the general public there was a strong association between agreeing to the “risk for complications”-argument and the “it is in the patient’s own interest”-argument *pro* the main claim (among physicians: [OR: 60.2 (95% CI: 8.1–448.3)]).

There was a strong association between physicians’ finding it right to make surgery conditional upon smoking cessation, and reporting that their trust in health care would increase if such policy was made standard procedure [OR 192 (95% CI: 56.6–708.7)].

In the *value-neutral*/*value-influenced* terminology presented in the Methods section above, 30.3% of physicians were classified as *value-neutral* and 69.7% were classified as *value-influenced.* In average 73.4% of the participating physicians stated that the presence of ‘medical risk’ was the most important argument for demanding smoke-cessation prior to surgery. But compared to the *value-neutral* physicians significantly more of the *value-influenced* physicians stressed the ‘medical risk-argument’ [79.4% (95% CI: 74.2–84.6) versus 61.0% (95% CI: 51.4–70.6)] (Chi-2 = 12.3; df = 1 and *p* < 0.001).

### Analysis of comments

The respondents were invited to provide comments to the main statement of whether it is right to make surgery conditional upon four weeks smoking cessation prior to and after surgery. Many comments, from physicians as well as from the general public, amounted to a qualification of this kind of condition-making. Such qualifying comments were found not only among those who opposed making surgery conditional upon smoking cessation, but also among those in favour of conditioning surgery. Thus, many commented that smoking cessation should be encouraged but not required, in effect supporting the line previously referred to as a strategy of “recommendation”. Comments referencing paternalistic reasons were more common among the population than among physicians, as were references to patients’ obligation to comply with the recommendations from health care and references to the virtue of being “a good patient”. Respondents from both groups raised “delimitation issues”, that is: a worry that if smoking cessation was made a condition for surgery, further difficult questions as to what else to demand would ensue. For an overview of content categories among physician respondents, please see Table 3.

**Table 3:** Shows comments to main statement grouped by respondent group and attitude (Number of comments in each comment category in brackets)

All physicians:		General public:	
Agree with the main statement	Disagree with the main statement	Agree with the main statement	Disagree with the main statement
Reject requirement (18) *“There are situations where demanding is impossible”; “Consider special cases”*	Reject requirement (22) *“Unethical to deny”; “Illegal to deny”*	Reference to risks^a^ (10) *“Unnecessary health risks”; “Wound healing and infections”*	Reject requirement (7) *“Right to recommend, wrong to require”; “It is not illegal to smoke”*
Reference to risks^a^ (15) *“Medically indicated”; “Smoking affects the outcome and therefore should be avoided”*	Delimitation issues (6) *“Who can determine where the line is to be drawn?”; “How about overweight patients?”*	Reject requirement (7) *“Too important to be made conditional”; “The physician needs to be a diplomat”*	Reference to risks^a^ (1) *“If the surgeon knows the patient will be much worse off”*
Methodological issues (10) *“This kind of surgery is not affected by smoking”; “8 weeks is too short”*	Methodological issues (3) *“Many other factors contribute to peri-operative risk”*	Paternalistic reasons (8) *“The patient should stop smoking forever”; “It is in the patient’s own best interest”.*	Delimitation issues (1) *“Who’ll decide what other life style related issues that should matter?”*
Reference to societal costs (9) *“Less costly to society”; “Complications are an economic burden to public health care”*	Reference to risks^a^ (1) *“Negligible risk”*	Obedience (3) *“The surgeon knows best”; “Patients should obey doctors’ orders”*	Methodological issues (1) *“How can the surgeon know the patient stopped smoking?”*
Miscellaneous (9) *“We have this policy”; “No reason not to”*		Reference to societal costs (5) *“Expensive not to take preventive measures”; “Expenses to the taxpayer”*	
		Reference to virtue (2) *“You should do what’s best”; “All should chip in”*	
		Miscellaneous (10) *“Seems reasonable”; “Should be done with all surgery”*	

^a^Some of the comments that make reference to risk seem to be focussing on surgery-related risk (“risk for complications”), whereas others refer to the general health risks associated with smoking

## Discussion

The main finding of this study is that among physicians and the general public alike, there was near universal support for making surgery conditional upon smoking cessation four weeks prior to and after surgery in a case with a 57 year old farmer in need of hip replacement surgery. Furthermore, this support was justified, in most cases, by reference to the risk for complications associated with smoking in the present setting.

Given the strong push to counteract smoking in society, the support for policies intended to decrease smoking is not surprising. However, it is interesting that a large majority of respondents in this study base their support on the patient’s alleged risk of surgical complications, although clinical research indicates that smoking only moderately increases peri-operative risk and that smoking cessation brings about only a moderate risk reduction. Thus, much of the discussion will be devoted to the question of why an activity with a moderate risk profile (smoking before and after orthopaedic surgery) gives rise to near uniform support for making surgery conditional upon its cessation. However, before trying to untangle that issue, we will briefly ponder another question: what, if anything, is signalled by the high prevalence among comments from the population and physicians alike of what seems to amount to an agreement with the main statement *but simultaneously* a rejection of the requirement strategy? To be sure, the number of respondents providing comments is limited, which means that no great conclusions can be drawn. Nevertheless, the pattern is so striking that it invites an attempt at interpretation. Most plausibly, the underlying logic is a support of what may be labelled a “soft requirement”. Respondents in favour of “soft requirement”, as we conceptualize it, support a general rule demanding smoking cessation prior to and after elective surgery, but hold that this rule should not be enforced in cases where patients fail to comply with smoking cessation. While we make no claim to have proved this, we suggest this possibility should be kept in mind in the interpretation of the material in this study and in possible subsequent studies.

Now we turn to the question of why there was such strong support for a policy (enforceable or not) of smoking cessation prior to surgery, despite the evidence from previous research that the possible direct health gain of such a policy is moderate. In regards to respondents from the general public, the most plausible answer is that these were influenced by the phrasing of the vignette where it was stated that the physician in charge warned of the medical risks, telling the patient that smoking infers an increased risk of difficult wound healing and infections after hip replacement surgery. However, things may be more complex when it comes to the physician respondents, as these likely had a preformed notion of the peri-operative risk before reading the vignette. Thus, we will explore two different possible explanations why a majority of physician respondents advocate that smoking patients should be denied hip replacement surgery because of the risks associated with this, although in fact the risks attributable to smoking in a case such as this do not, on their own, merit a blanket refusal of smoking patients for hip surgery.

In *risk overstatement*, the physician respondents believed that the risks attributable to smoking were larger than they are, much the same way as those respondents from the general public who were influenced by the phrasing of the vignette to overstate the risks. In *additional policy considerations*, the respondents may have had an accurate appreciation of the risk levels implicated by smoking, but, being led by additional policy considerations, concluded that all in all, a policy of demand for smoking cessation is desirable. Mixed versions of *risk overstatement* and *additional policy considerations* may also exist.

### Value impregnation?

Turning back to the initial discussion of value impregnation, we will now explore whether this phenomenon may have informed risk overstatement and/or additional policy considerations among physician respondents. Previous studies indicate that in settings characterised by strong moral sentiments, physicians tend to let their own values impregnate their factual assessments [25]. There was evidence of anti-smoker bias among respondents, including physicians, as evidenced by such stigmatizing comments as: “*It is stupid to smoke*” (physician responder); “*(non-smokers) smell better when one deals with them*” (physician responder). There was also evidence of paternalistic reasoning (see below). Thus, the present issue is such that one may expect the risk of value impregnation to loom large.

In this study, we found a significant difference between *value-neutral* and *value-influenced* physicians regarding the emphasis put on the “risk for complications”-argument in favour of making surgery conditional upon smoking cessation. Although theoretically both groups could misinterpret the risks, the most plausible interpretation here is that value-influenced physicians overstate the medical risks with surgery in the peri-operative setting. One possible reason for this may be a negative “halo effect” [29]. Physicians are well aware of the great general long-term health risks associated with smoking, and therefore may believe that the risk of peri-operative complications must be great also. If this is the case, the main ethical concern lies not in the judgment that surgery should be denied – since surgery should indeed be denied in any real high risk cases - but in the various grounds for the risk overstatement in the first place [30].

Suppose that some respondents correctly assessed the risk level as intermediate – putting the patient at the same risk level as, for instance, an older patient aged 77. Now, the 77 year old patient would presumably still be accepted for surgery, so if the 57-year old smoker in the vignette is denied surgery, some *additional policy considerations* must be at play. One such additional consideration may the personal conviction that smokers and others conceived to be responsible for their own bad health should, as a matter of principle, be down prioritized in health care (what we have previously referred to as “the responsibility principle”). Indeed some of the comments suggest support for the responsibility principle: “*Society should not pay if the patient does not heed doctors’ advice*” (general public responder), “*He can pay for his own surgery!*” (physician responder); “*Why should society pay for risks that the patient causes himself*” (general public responder). Our method does not make it possible to directly assess the levels of risk overstatement or support for additional policy considerations, nor to pry the two phenomena apart in the individual case. Nevertheless, this principled discussion goes some way to explain why there is such strong support for smoking cessation prior to and after surgery.

### Paternalism

If one agrees to the main claim because of the *general health gain* to be expected with smoking cessation, even outside of the effects related to the surgery at hand, this constitutes a prime additional policy consideration. Although few respondents named it the most important argument, 65.8% of physicians and 72.0% of the general public agreed with the argument “Because it is in the patient’s own interest to stop smoking altogether”. This is, however, an arguably paternalistic view as it suggests that the patient, despite his declared preferences, does not understand his own good. As such this view may have motivated some of the respondents’ moralising judgments. The aim for long-term smoking cessation may also inform the previously quoted opinion from Thomson et al.: “the preoperative period might be a window for smoking intervention” (7). On this view, the overarching aim is to stop patients from smoking for a number of reasons, and helping (“recommendation”) or forcing (“requirement”) them to stop before surgery is an effective way of doing so. Some comments also indicated paternalistic attitudes: *“What the patient thinks is not as important as what the doctor thinks”* (respondent from the general public); “*Rational arguments don’t bite on addicts”* (physician respondent); *“If the patient does not want to give up smoking, this proves he has not understood the consequences”* (physician respondent)*.* It is further interesting to note that there was a strong association between support for the “it is in the patient’s own interest”-argument and the “risk for complications”-argument. This indicates that some respondents may indeed overstate the risk for complications due to their desire to make the patient stop smoking altogether. All in all, there are indications that paternalistic attitudes may underlie the overwhelming support of smoking cessation prior to and after surgery in our material.

### Further problems with the “suitable window” image

As we have already mentioned, the “suitable window of opportunity” [7] imagery may serve as a vehicle for paternalism. We would like to present a further argument why this imagery may be problematic. If the point that the health care professionals wish to drive home is not mainly related to the surgery itself but to the patient’s future health, then why is the pre-operative period seen as preferred over any other situation when the patient encounters health care? To our minds, there is no particular vantage point from which it is a priori more justified to recommend smoking cessation. Therefore we are concerned that insistence on using this precise moment to discuss the patient’s smoking habits may stem from a paternalistic wish to reap health benefits from the patient’s vulnerable situation at that moment. The patient may experience both pre-surgical anxiety and a fear of being denied surgery, which puts the patient in a weak situation relative to the physician. On the one hand such a position may be instrumentally advantageous if the physician wants to influence or persuade the patient to do something, but on the other hand such “aggressively benevolent” behaviour seriously threatens the patients’ autonomy [31]. Thus, from an ethical standpoint the preoperative situation may indeed prove to be not such a suitable window of opportunity after all.

There is an analogy between the above reasoning and the general discussion on the use of fear in clinical communication. Resorting to “fear appeals” or “fear mongering” – using or intentionally causing fear and anxiety in patients to make them change their behaviour – is arguably in conflict with the non-maleficence principle [32]. More specifically, if for paternalist reasons the physicians exaggerate the dangerousness of smoking (in the actual case), this is contrary to the physician’s duty, derived from respect for patient autonomy, to provide adequate information. As put by American medical ethicists Beauchamp and Childress: “lying, withholding information and misleading by exaggeration…all compromise autonomous choice” [33]. Indeed, even the medical jargon used may be an instrument of disguised paternalism. Terms such as “medical risk” or “medical indication” may perform as a sort of trump card, as patients lack sufficient understanding of the vagueness of these terms [34]. It is our general conviction that the way physicians use the word ‘risk’ and communicate risk-estimations is an area deserving of further ethical and empirical investigation. In sum, although there are negative health effects with smoking in some peri-operative settings and most certainly in the long run, this does not justify physicians to make exaggerated claims of the present risks.

### The merits of “recommendation” versus “requirement” strategies

Our own view, which is heavily informed by considerations of patient autonomy, is that the only acceptable strategy is a carefully worked out strategy of “recommendation” of smoking cessation prior to surgery. This, to our minds, is the best way of acknowledging the limited risk increase due to smoking in the peri-operative setting, without falling prey to the ethical risks discussed above. If our position is accepted, it means that health care staff must make an effort to keep all information as correct and free from “fear-mongering” as possible, and that they discern between the possible health gains that relate to the elective surgery at hand and those that relate to long term effects, respectively. We are aware, however, of one complication of the “recommendation” strategy, which it shares with the “requirement” strategy. The empirical studies show that four or even eight weeks of smoking cessation are necessary to minimize surgery-related risk. On top of that, qualified behavioural counselling, if necessary, may itself take some time. Thus, it may be that the “recommendation” strategy actually postpones surgery up to several months as compared to making no difference between smoking patients and other patients at all. Can the strategy of “recommendation” for smoking cessation still be accepted? Yes, but only as long as the negative effects of postponing surgery are offset by the (expected) positive effects of smoking cessation. In the case where the expected positive effects are small, and the “costs” in terms of suffering by waiting are large, then the “recommendation” strategy fails. In the reverse case, it may be that smokers who refuse to join a smoking cessation programme reach the surgery table before those that want to try for smoking cessation. This we do not see as a downfall of our view but as a reasonable effect of respecting patient autonomy.

That being said, we turn now to the “requirement for attempt” and the “requirement for cessation” strategies, both of which we find unacceptable. To be more precise, there is one fully legitimate subset of “requirement for cessation”, namely any case that falls under the principle of non-malevolence. If the medical risks of surgery exceed the expected gains, then surgery should not be performed. In the words of one respondent: *“It (=whether or not to demand smoking cessation) should be related to how large risks the smoking imposes in each particular case”*. This, of course, has nothing to do with smoking per se, but applies to *any* strong risk factor. But as long as the risk increase does not exceed what would permit surgery in other patient groups, we find no reason to accept the “requirement for cessation” strategy.

The strategy of “requirement for attempt” may seem to be an acceptable middle ground between the “recommendation” and the “requirement for successful cessation” strategies, since it appears more respectful to the patient’s autonomy than the “requirement for cessation” strategy. However, this appearance is deceptive. To summarize, the “requirement for attempt” refers to the position expressed in this way by one physician respondent: “*If the patient tries but can’t stop, then s/he should be accepted anyway*”. Now, this readiness to accept the patient for surgery regardless of the success of smoking cessation means that adherents of a “requirement for attempt” implicitly downplay the medical risks with surgery for a smoking patient. In principle, this is unacceptable. If the medical risks associated with smoking were indeed grave, then, in line with the previous reasoning, surgery should not be performed as long as the patient smokes. In that case, nothing short of smoking cessation is sufficient as a requirement for surgery. Thus, the “requirement for attempt” is a purely paternalistic attitude; it is trying to take advantage of the previously mentioned “window of opportunity” to reap long term benefits [7]. The strategy, therefore, is more problematic than first intuition may indicate.

### Strengths and weaknesses

The strengths of this study lies in part in its semi-experimental design – the case vignette model is known to give robust data, especially regarding external validity [35]. Thus, the main finding likely has good generalizability. Furthermore, to our knowledge this is the first large study of the attitudes among Swedish physicians to making surgery conditional upon smoking cessation prior to surgery. The distinction between value-influenced and value-neutral physicians has been used in other studies, and appears to capture an aspect of clear ethical importance [36].

However, the study also has limitations. The main limitation is that it included one vignette only. Therefore, it is hard to assess how the answering patterns were influenced by the framing of the case presentation. If the study were repeated, it would be interesting to use several case presentations to compare answering patterns across these. It would also be interesting to explicitly spell out varying levels of expected smoking-related risk increase across several case presentations, to eliminate the possible bias resulting from respondents’ differing conceptualization of the risk for complications. It would also have been of value to provide questions to assess support for “recommendation” vs “requirement” strategies directly, and/or to measure the level of negative attitude towards smokers and correlate this with the displayed attitudes towards making surgery conditional upon smoking cessation. An idea for a whole other study would be to conduct a qualitative analysis of physicians’ reasoning across varying levels of peri-operative smoking-related risk.

## Conclusions

This vignette study showed that physicians as well as members of the general public favour making surgery conditional upon smoking cessation prior to surgery, although analysis of the comments indicated that support may in fact be for the weaker “recommendation” strategy. The respondents justified their standpoint mainly by reference to increased peri-operative complication risk connected with smoking, but there were indications that paternalistic, responsibility-based and stigmatizing attitudes towards smokers also influenced the respondents. We thus argue that value impregnation might play a role in exaggerating the risk for complications as well as providing additional policy considerations in regards to peri-operative smoking cessation recommendation. Moreover, we argue that “requirement” strategies of smoking cessation are ethically flawed.

In the light of prevalent anti-smoker bias, we recommend that physicians carefully examine their assessment of smoking patients’ surgical risks to prevent influence from ethically unacceptable attitudes. Smokers deserve the same kind of risk assessment as other patients do. We further recommend that physicians address the topic of smoking with their patients in all suitable situations, not only or even predominantly in situations where the patient is dependent upon help from health care.

In the words of one respondent, we conclude: it is right to recommend smoking cessation prior to surgery, but wrong to require it.

## Additional file

Additional file 1: Questionnaire (English) This is an English translation (made by the first author) of the questionnaire sent out to all respondents. The questionnaire version sent out to physicians included one additional question (regarding field of medical specialization) as opposed to the version sent out to members of the general public. This question is marked with an asterisk (*) for clarity. (DOCX 17 kb)

## Abbreviations

CI: Confidence interval
GPs: General practitioners
OR: Odds ratio
