# Supplementary material for: “Right to recommend, wrong to require”- an empirical and philosophical study of the views among physicians and the general public on smoking cessation as a condition for surgery
Source: BMC Med Ethics. 2018 Jan 8;19:2. doi: 10.1186/s12910-017-0237-2 (PMC5759185; doi:10.1186/s12910-017-0237-2)
Supplement: Additional file 1: — Questionnaire (English) This is an English translation (made by the first author) of the questionnaire sent out to all respondents. The questionnaire version sent out to physicians included one additional question (regarding field of medical specialization) as opposed to the version sent out to members of the general public. This question is marked with an asterisk (*) for clarity. (DOCX 17 kb) [file 12910_2017_237_MOESM1_ESM.docx]

A 57 year old farmer has long time pains from his right hip. Medical examinations show osteoarthritis. The patient’s profession is physically demanding with plenty of movement and lifting. The hip is bothering the patient and he has been told that a hip replacement would make his situation easier.

However, the surgeon has told the patient that in order to qualify for surgery, he needs to stop smoking four weeks before and four weeks after the surgery. The patient is a long time pipe smoker and considers this a major source of life quality.

According to the surgeon, smoking infers an increased risk of difficult wound healing and infections after hip replacement surgery. The surgeon recommends the patient to enlist in a smoke cessation programme, so that he can be smoke free even in the long run. The patient declines this – he does not want to give up smoking.

Neither the surgeon or the patient will change his mind, and the patient turns to a private health care provider and has his surgery there. Because of this, he has to pay the costs of surgery and post surgical care out of his own pocket (which he would not otherwise have had to do)

*After having read the above, please consider the following statements. Indicate your best answer by a check mark below:*

1. It is right, in a case such as this, to make the planned surgical procedure conditional upon the patient’s stopping smoking 4 weeks prior to and 4 weeks after surgery.
   □ agree completely
   □ agree to a large extent
   □ disagree to a large extent
   □ disagree completely.

   Comments:

*Below you find two arguments in support of (and later also two arguments against) the surgeon’s conditions. Please indicate whether you agree with the arguments.*

1. Because of the risk for complications the patient in a case such as this should accept four weeks smoking cessation prior to and after surgery
   □ agree completely
   □ agree to a large extent
   □ disagree to a large extent
   □ disagree completely.

   Comments:
2. Because it is in the patient’s own interest to stop smoking altogether it is good that partaking in a smoking cessation programme is made mandatory to qualify for surgery
   □ agree completely
   □ agree to a large extent
   □ disagree to a large extent
   □ disagree completely.

   Comments:
3. If you have other arguments supporting the surgeon’s conditions, please state them here:

*Here are two arguments against the surgeon’s conditions:*

1. If a patient, as in the case above, is well informed about the risks the surgeon should go on with surgery
   □ agree completely
   □ agree to a large extent
   □ disagree to a large extent
   □ disagree completely.

   Comments:
2. If a patient who is well informed about the risks, as in the case above, states that smoking is important to his/her life quality, then the surgeon should go on with surgery
   □ agree completely
   □ agree to a large extent
   □ disagree to a large extent
   □ disagree completely.

   Comments:
3. If you have other arguments against the surgeon’s conditions, please state them here:
4. Which of the above arguments do you think is the most important in this situation?
5. If surgeons in cases like this were to routinely make surgery conditional upon four weeks smoking cessation prior to and after surgery, this would affect my own trust in health care thus:
   □ my trust would decrease
   □ my trust would not be influenced
   □ my trust would increase

   Comments:

Now for some questions about you:

I am □ a man □ a woman Age:

I smoke □ yes □ no

I have previously been a smoker □ yes □ no

My trust in health care is currently
□ very high
□ moderately high
□ moderately low
□ very low

What is your medical speciality?*
□ Family medicine
□ Orthopaedic surgeon
□ Other

Comments:

Other comments:

Thank you for participating!

**This question was included only in the questionnaires sent out to physician respondents*
